# The best defense is a good offense: Anti‐predator behavior of the common octopus (*Octopus vulgaris*) against conger eel attacks

**DOI:** 10.1002/ece3.11107

**Published:** 2024-03-19

**Authors:** Beatriz Salvador, Miguel Cabanellas‐Reboredo, Manuel E. Garci, Ángel F. González, Jorge Hernández‐Urcera

**Affiliations:** ^1^ ECOBIOMAR Research Group Institute of Marine Research (IIM‐CSIC) Vigo Spain; ^2^ National Center Spanish Institute of Oceanography CSIC, Balearic Islands CO Palma Spain

**Keywords:** cephalopods, conger eel, defense strategy, octopus, survival tactics

## Abstract

We present the description of defensive behavior in wild *Octopus vulgaris* against conger eel (*Conger conger*) attacks based on three video sequences recorded by recreational SCUBA divers in the eastern Atlantic off the coast of Galicia (NW Spain) and in the Cantabrian Sea (NW Spain). These records document common traits in defensive behavior: (1) the octopuses enveloped the conger eel's head to obscure its view; (2) they covered the eel's gills in an attempt to suffocate it; (3) they released ink; (4) the octopuses lost some appendages because of the fight. In the third video, the octopus did not exhibit the defensive behavior described in the first two videos due to an inability to utilize its arms in defense, and the conger eel's success in capturing octopuses is discussed. Additionally, both the cost that the octopus could face by losing some arms during the fight and whether the experience it acquires can be an advantage for future encounters are analyzed. The defensive behavior exhibited by octopuses in this study highlights their ability to survive in a hostile environment and serves as an example of the extensive repertoire of anti‐predator strategies employed by these cephalopods.

## Graphical abstract

The first description of defensive behavior in wild *Octopus vulgaris* against conger eel attacks is presented from three observations made in the Atlantic Coast of Galicia (NW Spain) and Cantabrian Sea (NW Spain), which were filmed by scuba divers. The defensive behavior shown by octopuses in this work can help to understand the evolution of anti‐predator strategies in cephalopods.

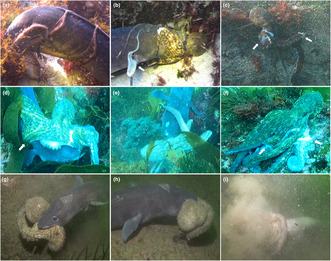

## 1 INTRODUCTION

Octopuses are characterized by their complex behavior, exhibiting a wide diversity of defense strategies against different predators comparable to those of some higher vertebrates (Amodio et al., 2019; Packard, 1972). The most commonly used strategies are those for avoiding predators such as camouflage, crypsis, or flight (Hanlon et al., 1999; Hernández‐Urcera et al., 2019; Josef et al., 2012). They are also capable of autotomy, in which they sacrifice one or more arms to a predator in order to escape from capture (Jaitly et al., 2022). For this reason, it is very common to find octopuses with missing or partially amputated arms (Hanlon & Wolterding, 1989; Nesis, 1982).

Octopuses are thought to be especially vulnerable to predation by eels, which may use olfactory cues to find them (Forsythe & Hanlon, 1997; Lane, 1957; MacGinitie & MacGinitie, 1968; Randall, 1967). In their predatory behavior, eels utilize a combination of chemosensory cues and visual acuity to locate and hunt octopuses. Upon detecting chemical signals emitted by octopuses or discerning their visual presence, eels employ stealth and agility to close in on their prey swiftly (Lane, 1957). Once in close proximity, eels rely on their muscular bodies and sharp teeth to subdue and capture octopuses for consumption (Ambrose, 1988).

Conger eels (*Conger conger*) are one of the major predators of cephalopods in rocky areas of the Northeast Atlantic and Mediterranean Sea (Cau & Manconi, 1984), with octopuses being the second most frequent prey as determined by stomach content analysis (Xavier et al., 2010). The notable larger size of conger eels when compared to the octopuses they capture (Xavier et al., 2010) makes it unlikely that the octopus can escape the eel's attack. Nevertheless, octopuses exhibit sophisticated defensive behaviors to escape these predators that were successful in two of three recorded instances. We herein describe this defensive behavior of *Octopus vulgaris* against conger eel's attacks on three occasions in rocky areas off the coast of northwest Spain.

## 2 MATERIALS AND METHODS

Encounters between conger eels (*Conger conger*) and octopuses (*O. vulgaris*) were observed three times in different locations along the Northern Spanish coasts. All observations, made by SCUBA divers, were recorded using underwater video cameras (Canon G9 within Canon WP‐DC34 housing and GoPro Hero9 Black in observations 1 and 2, respectively; and Sony RX100 within Nauticam housing in observation 3) and documented by citizen scientists before being relayed to the Cephalopod Behavior Lab at the Institute of Marine Research (IIM‐CSIC). The first video was recorded on rocky substrate around Ons Island (Pontevedra; Atlantic Ocean; NW Spain; 42°22′52″ N, 8°55′5″ W) on November 16, 2008 (around 10 am) at 12 m. The second video was recorded on a rocky substrate in Las Minas Islet (Asturias; Cantabrian Sea; NW Spain; 43°33′21″ N, 6°31′19″ W) on July 2, 2022 (around 1 pm) at 15 m. The third video was recorded on a rocky substrate in San Martiño Island (Cíes Islands; Atlantic Ocean; NW Spain; 42°14′02″ N, 8°54′09″ W) on February 9, 2023 (around 11 am) at 20 m. The size of the octopuses and congers was estimated from the video recordings, comparing them with objects of known dimensions (e.g., mussels and other bivalve shells). The octopus's weight was estimated from dorsal mantle length value, using the formula of Hernández‐García et al. (2002).

## 3 RESULTS

### 3.1 First record

**FIGURE 1:**
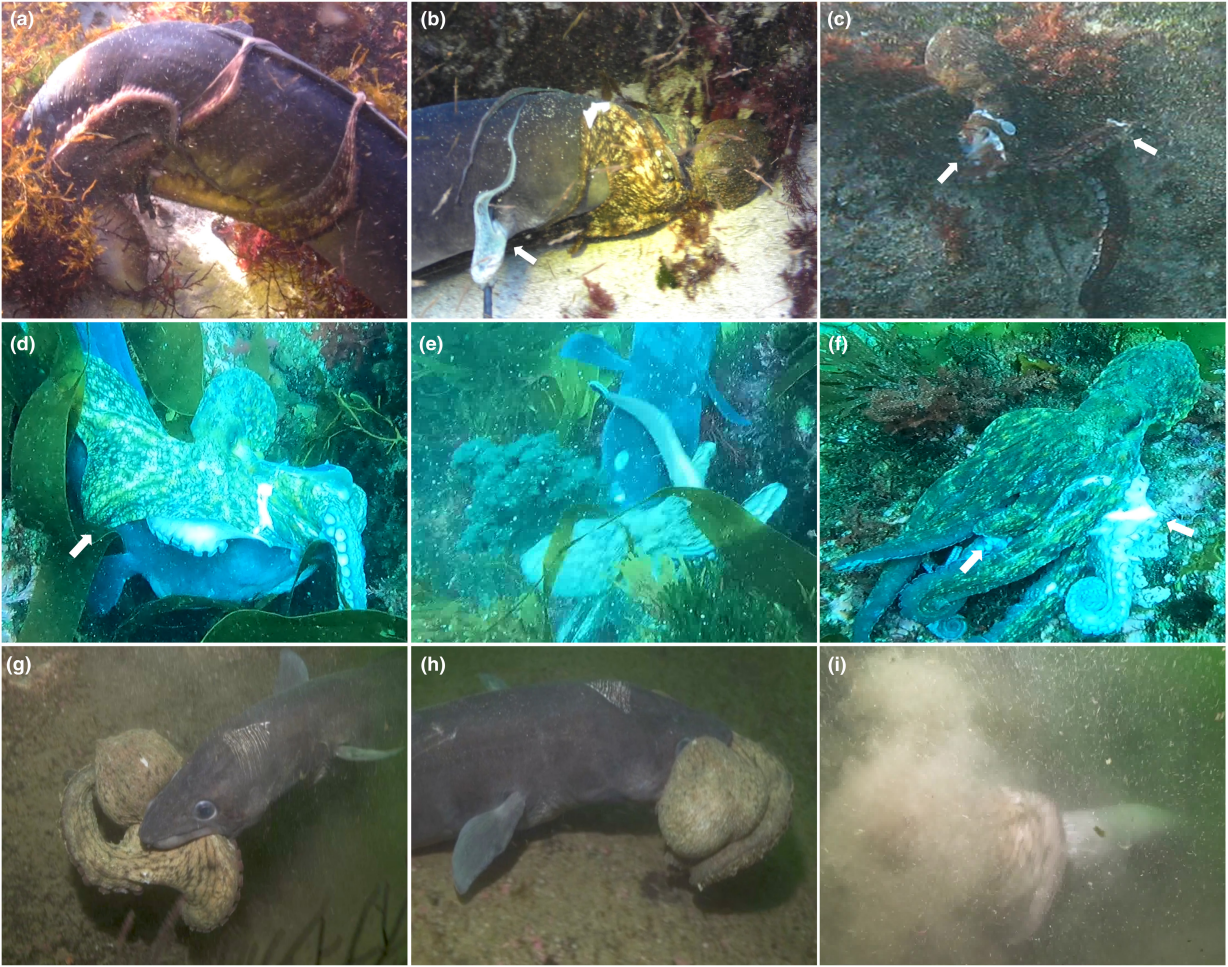
Frames extracted from the videos recorded at Ons Islands (a–c), Las Minas Islet (d–f), and San Martiño Island (g–i). (a) *Octopus vulgaris* envelops a conger eel and covers its gills. (b) *O. vulgaris* with one of its arms perforating a conger eel's gill (white arrow). (c) *O. vulgaris* without two amputated arms (white arrows) after the altercation. (d) *O. vulgaris* trying to suffocate a conger eel by covering its gills (white arrow). (e) *O. vulgaris* releasing ink while the eel shakes its head to free itself. (f) *O. vulgaris* with two amputated arms (white arrows) after the altercation. (g, h) *O. vulgaris* caught by a conger eel. (i) The conger eel shaking its head and spinning around, despite a lack of defensive response from the octopus.

This record (Video S1) involved an individual *O. vulgaris* of about 1800 g total body weight (BW) captured by a conger eel of approximately 180 cm total body length (BL). During the attack, the octopus occluded the conger eel's eyes with its arms to potentially obscure its vision (Figure 1a) despite having three arms enclosed within the mouth of the predator. Following this, the octopus began to cover one of the conger eel's gills, probably in an attempt to suffocate it while one of its bitten arms perforated another gill slit (Figure 1b). This state persisted for a few minutes until the conger eel shook its head and spun, freeing itself from the octopus' arms. Surprisingly, once the eel's spinning ceased, the octopus moved back into its former position on the eel's head. After several seconds, the divers separated them, and both animals rapidly moved away, with the octopus inking as it jetted away. It had lost two arms and another one was semi‐amputated after the fight (Figure 1c).

### 3.2 Second record

In Video S2, an individual *O. vulgaris* of about 2000 g BW had three of its arms bitten by a conger eel that was approximately 140 cm BL. The octopus encircled the conger eel's head with its remaining arms and tried to suffocate it by occluding its gills (Figure 1d). The eel remained static for a minute and a half before it began to shake its head, freeing itself from the octopus' arms. However, the octopus then released its ink (Figure 1e) and adopted the same position again. After a minute, the eel released the octopus from its mouth and it escaped. The octopus survived the fight but lost one arm while two others were semi‐amputated (Figure 1f). Additionally, it appeared that the eel suffered the loss of an eye during the altercation.

### 3.3 Third record

This observation (Video S3) was of an individual of *O. vulgaris* of about 1500 g BW caught by a conger eel of approximately 130 cm BL. The eel bit and held the octopus by its mid‐body between the base of the arms and the head/mantle, preventing any movement (Figure 1g,h). In reaction to the diver, the eel swam away with the octopus in its mouth for around 60 s. At the end of the observation, the eel began to shake its head and spin around while the octopus remained still (Figure 1i). In this instance, the octopus did not show the defensive behavior seen in the other videos, but it could be that it was stunned or dying when the diver started recording the altercation.

### 3.4 Consistent features across the defensive behavior of *O. vulgaris*

Defensive behavior was observed during the two first altercations. In both instances, the octopus was smaller than its predator. Defensive behavior followed a consistent pattern: First, the octopus began to encircle the conger eel's head to obscure its view. Next, it tried to suffocate the eel by covering its gills. On both occasions, the octopus released ink, and both altercations ended in the loss of appendages for the octopus. In the third video, there is no expression of the defensive behavior described in the first two instances.

## 4 DISCUSSION AND CONCLUSIONS

Conger eels are a constant threat to octopuses since they inhabit cavities (Glaeser & Abed‐Navandi, 2023) often investigated blindly by foraging octopuses (Hanlon & Messenger, 2018). Furthermore, the morphology and sensory capabilities of conger eels allows them to reach octopuses in their dens (Forsythe & Hanlon, 1997). The video sequences described herein represent the first video records of defensive behavior against conger eels' attacks in wild *O. vulgaris*. Our observations suggest that the success of the conger eel depends on how it attacks the octopus. This hypothesis arises from the fact that the octopuses in the first and second videos escaped, while that in the third did not. If the conger eel captures the octopus only by its arms, the octopus has a higher chance of escaping, since it could use the unbitten arms to defend itself, as occurs in the previously described defensive behavior. By contrast, if the octopus is captured on the head or mantle, it is unable to use its arms to defend itself and the conger eel performs a characteristic rotational movement to tear the prey into smaller pieces, known as rotational feeding (Mehta et al., 2010). Ripping apart the octopus could help the conger eel avoid the risk of asphyxiation, as seen in dolphins feeding on whole octopuses (Stephens et al., 2017). This rotational movement observed also as a defense mechanism in other anguillid eels (Musumeci et al., 2014), was potentially employed by the conger eels in the first two records in an attempt to free itself from the octopus's arms.

A similar pattern of fighting predators off has been observed against pajama sharks (*Poroderma africanum*; Jeffs & Brownlow, 2017) wherein octopuses employ their arms to disrupt the shark's breathing by inserting them into the shark's gills, facilitating their own release when captured. Furthermore, octopuses are known to employ an asphyxiating ‘constricting’ strategy also as an aggressive tactic, both towards conspecific in the context of sexual cannibalism and towards other species of octopus (Huffard & Bartick, 2015). In this way, they prevent water from flowing to their opponent's gills, which is the same purpose for which they perform the behavior described in this work.

The octopuses in the first two videos released ink during the altercations. While in the first video, the octopus releases ink after escaping, potentially to deter the conger eel from pursuing it, in the second video, the octopus releases ink while still being held by the conger eel. The ink released by an octopus contains compounds that can disrupt the sensory perception of predators like the conger eel, affecting their ability to locate prey accurately (Derby, 2014). This defense mechanism of the octopus interferes with the predator's olfactory and visual senses, providing an opportunity for the octopus to escape predation.

The presented records in this article highlight the cost incurred by the octopus during its defensive response against conger eel attacks, which serves as a last‐ditch effort when already captured. In both the first and second cases, the octopus loses a pair of arms during the altercation. Given the high degree of autotomy of octopus arms and suckers (Nesher et al., 2014), it cannot be ruled out that one of the arms was autotomized. If the suckers of an amputated arm remain attached to the conger eel (internally or externally), then the octopus could increase its chances of surviving the attack. According to Fossati et al. (2015), the whole arm of an octopus kept in laboratory conditions takes approximately 42 days to regenerate all the lost structures. As a result, the octopus faces the cost of being without these arms for the duration of regeneration and the concomitant less of predatory and defensive abilities (e.g., hunting; Mather, 1998). Furthermore, octopuses show a preference for some of their arms (Byrne et al., 2006a, 2006b), so losing one of these preferred arms would still pose a greater vital challenge. However, it can survive this regeneration period, which has already been reported through observations in the wild (Hanlon & Messenger, 2018), with divers frequently observing octopuses with arms in the process of regeneration.

Additionally, the altercation may be a learning experience that puts octopuses at an advantage in future encounters with similar predators. Octopuses have been shown to exhibit extraordinary long‐term memory and associative learning (Hochner, 2010; Schnell et al., 2021), mainly in activities related to sight and touch (Hochner, 2008), lasting weeks and even months (Schnell et al., 2021). Through these capabilities, octopuses can effectively assess threats and develop strategies to mitigate risks posed by predators. This ability to learn from past experiences enhances their overall success in navigating complex marine ecosystems, where predator–prey interactions play a critical role in shaping ecological dynamics (Zarella et al., 2015).

In conclusion, this study sheds light on the intricate defensive strategies of octopuses when confronted with conger eels, employing a variety of defense mechanisms to evade predation. The observation of defensive behavior in the face of conger eel attacks provides valuable insights into predator–prey dynamics in marine ecosystems. However, limitations such as the small number of observations and incomplete altercation videos underscore the need for further research to comprehensively understand octopus defense strategies. Future studies could explore larger sample sizes and controlled experimental settings to elucidate the nuances of octopus defensive behaviors and their ecological implications. Ultimately, understanding octopus defensive behaviors contributes to our broader understanding of predator–prey interactions and ecosystem dynamics in marine environments.

## AUTHOR CONTRIBUTIONS

**Beatriz Salvador:** Conceptualization (equal); formal analysis (equal); methodology (equal); visualization (equal); writing – original draft (lead). **Miguel Cabanellas‐Reboredo:** Conceptualization (equal); supervision (supporting); writing – review and editing (equal). **Manuel E. Garci:** Investigation (supporting); methodology (supporting); writing – review and editing (supporting). **Ángel F. González:** Conceptualization (equal); funding acquisition (equal); supervision (equal); writing – review and editing (equal). **Jorge Hernández‐Urcera:** Conceptualization (equal); formal analysis (equal); funding acquisition (equal); investigation (equal); methodology (equal); supervision (lead); validation (equal); visualization (equal); writing – review and editing (equal).

## FUNDING INFORMATION

B.S. was supported by a Jae Intro fellowship granted by CSIC (JAEINT_22_0220). J.H.U. and M.C.R. were supported by two Juan de la Cierva's post‐doc research grants (IJC‐2020‐043701 and IJC‐2019‐038852, respectively; Ministerio de Ciencia e Innovación, Spain). ECOSUMA Project (PID2019‐110088RB‐I00) partially supported this study.

## CONFLICT OF INTEREST STATEMENT

The authors declare no competing interests.

## Supporting information


Videos S1–S3

## Data Availability

Videos of the three observations of conger eel attacks on octopus in Spain (Videos S1–S3).
