# Supplementary material for: The best defense is a good offense: Anti‐predator behavior of the common octopus (Octopus vulgaris) against conger eel attacks
Source: Ecol Evol. 2024 Mar 19;14(3):e11107. doi: 10.1002/ece3.11107 (PMC10951491; doi:10.1002/ece3.11107)
Supplement: Supplementary file 1 — Videos S1–S3 [file ECE3-14-e11107-s001.zip › Video captions.docx]

Video 1: Observation recorded in Ons Islands (Atlantic Ocean; NW Spain)

Video 2: Observation recorded in Las Minas Islet (Cantabrian Sea; NW Spain)

Video 3: Observation recorded in San Martiño Island (Atlantic Ocean; NW Spain)
